# Bi_2_O_3_ Nanoparticles Decorated Carbon Nanotube: An Effective Nanoelectrode for Enhanced Electrocatalytic 4-Nitrophenol Reduction

**DOI:** 10.3389/fchem.2020.00325

**Published:** 2020-05-08

**Authors:** Raviraj P. Dighole, Ajay V. Munde, Balaji B. Mulik, Bhaskar R. Sathe

**Affiliations:** Department of Chemistry, Dr. Babasaheb Ambedkar Marathwada University Aurangabad, Aurangabad, India

**Keywords:** 4-NP, synergetic effect, Bi_2_O_3_@MWCNTs nanocomposite, environmental pollutant, electrochemistry

## Abstract

4-Nitrophenol (4-NP) is present in most industrial waste water resources as an organic pollutant, and is a highly toxic and environmentally hazardous pollutant. Herein, we report that bismuth oxide (Bi_2_O_3_) decorated multi-walled carbon nanotubes (Bi_2_O_3_@MWCNTs) are the most prominent electrocatalyst for 4-NP electroreduction in acidic conditions. The electrocatalyst is synthesized by a simple chemical reduction method using ethylene glycol as a capping agent. The synthesized Bi_2_O_3_@MWCNTs electrocatalyst has been well-characterized by Fourier-transform infrared (FT-IR) spectroscopy, transmission electron microscopy (TEM), X-ray diffraction (XRD), and Raman spectroscopy. Bi_2_O_3_@MWCNTs have a cubic structure which is confirmed by XRD. TEM imaging reveals Bi_2_O_3_ NPs are ~2 nm in size, are grown on MWCNTs and that these nanoparticles are active toward 4-NP electroreduction. The electrochemical studies by cyclic voltammetry measurements show that the Bi_2_O_3_@MWCNTs electrocatalyst can sense 4-NP at a very low potential i.e., −0.17 vs. saturated calomel electrode (SCE). Furthermore, electroanalytical parameters like scan rate and concentration dependence were studied with electrochemcial impedance spectroscopy (EIS) and the effect of pH on cathodic current was examined under experimental conditions. The lower limit of detection (LOD) was found to be 0.1 μM for the Bi_2_O_3_@MWCNTs nanomaterial and is excellent toward 4-NP. The present study has applications for reducing water pollution and for sorting out related issues.

**Graphical Abstract:**
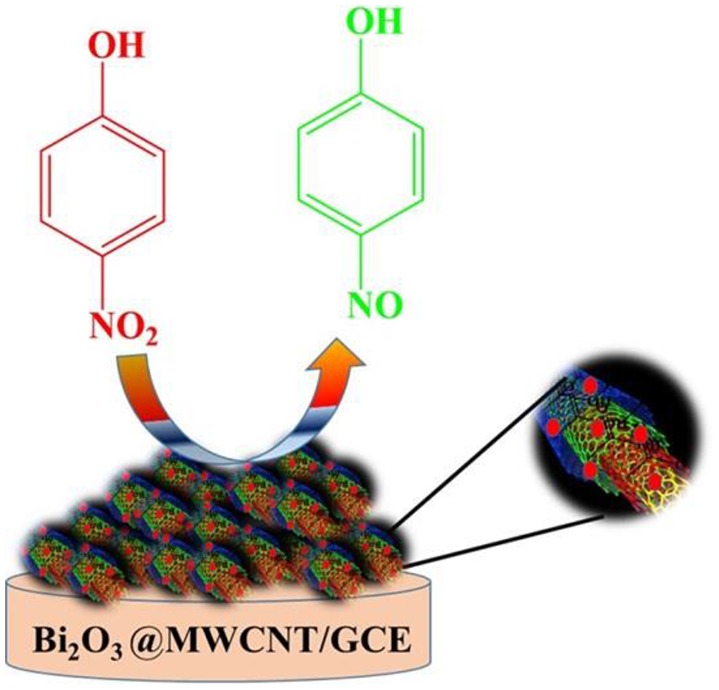
Electrochemical Determination of 4-Nitrophenol on Bi_2_O_3_@MWCNT Nano electrode.

## Introduction

4-Nitrophenol (4-NP) is an important compound as it is a precursor in the manufacturing processes of drugs, pesticides, dyes, fungicides, insecticides, explosives, and is used to darken leather (Li et al., 2012; Veerakumar et al., 2015; Rajkumar et al., 2018; Wu et al., 2018). However, it is a hazardous compound and has been shown to be present in industrial waste water and in fresh water where it comes from agricultural field run-off due to the degradation of organo-phosphorus pesticides (Wu et al., 2018). 4-NP is responsible for soil and water pollution because of its high level of toxicity. Due to this, the US Environmental Protection Agency (US EPA) has listed 4-NP as a priority pollutant. According to the US EPA the acceptable limit of 4-NP in potable water is only 60 ppb. Consuming even a small amount leads to acute effects in humans such as nausea, headache, drowsiness and cyanosis (Bose and Ramiah, 2017). It is even more dangerous as it can act as carcinogenic and mutagenic agent (Rajkumar et al., 2018). So there is an urgent need for the detection of 4-NP.

In the literature there are various methods employed for the determination of 4-NP *viz*. Belloli et al. (1999) determined 4-NP levels through a simple isocratic HPLC approach using seven columns, where the columns contained silica based C_18_ material. In this process a SiOH cartridge was used, which was treated with methyl chloride, dried with helium gas and stored at a low temperature. Sampling took 4 h. Guo et al. (2004) performed the separation and determination of phenol isomers by using capillary zone electrophoresis with methanol as an additive, it is an efficient method and about 10^5^ theoretical plates per meter was achieved. Zhong et al. (2011) reported a method of detection in waste water, it is a 3-step process comprised of sample preparation, determination by GC-MS and subsequent analysis. Lin et al. (2013) proposed spectrophotometric detection, in which 4-NP was pre-concentrated with 1-hydroxy-2-naphthoic acid modified TiO_2_, under optimum conditions more than 98% adsorption can be achieved by this method. Each method has certain limitations associated with it, such as the detection limit, sensitivity, selectivity, heavy instrumentation requirements, use of expensive gases, pretreatment etc. Moreover, the electrochemical method is a prominent technique for the detection of effluents like 4-NP as it is a rapid, cost effective, easy to operate, highly sensitive, and most importantly on-site measurement, that requires mild reaction conditions and has a low limit of detection. These factors make it a better alternative compared to the rest of the methods (Sun et al., 2012; Lin et al., 2013; Barman et al., 2017). Due to these factors, many electrochemical sensors have been reported for the determination of 4-NP. Chemically modified electrodes proved to be the best electrocatalysts as they increase the rate of reaction, provide a high surface area, have good stability and selectivity. A literature survey revealed that graphene based electrocatalysts (Li et al., 2012), metal and metal oxide electrocatalysts (Barman et al., 2017; Singh et al., 2017), metal-free electrocatalysts (Rajkumar et al., 2018), and their composites were all briefly studied as methods to determine 4-NP concentration.

There are several materials used as conductive substrates on which metal nanoparticles are anchored and grown including graphene (Chen et al., 2011), carbon nanotubes (Umar et al., 2016), Fullerene C_60_ (Li et al., 2019), graphitic carbon nitride (Liu et al., 2018; Hassannezhad et al., 2019) and conducting polymers like polyaniline (Wang G et al., 2018). Among these, carbon nanotubes have drawn attention since their inception in 1991, because of their unique properties including excellent electrical conductivity, large surface area, high electron density, exceptionally high mechanical and chemical stability, all of which makes them an ideal supporting material (Umar et al., 2016; Liu et al., 2018). Furthermore, the Ni based nanocomposite, MgFe_2_O_4_ NPs proved to be a good electrocatalyst for 4-nanoparticles (NPs) reduction (Baby et al., 2019; Mejri et al., 2019). Carbon nanotubes decorated with metals like Pt, Pd, Ag, monometallic-Au, and bimetallic-Au show excellent electrocatalytic activity toward nitrophenols (Liu et al., 2015; Umar et al., 2016; Sun et al., 2017; Dhanasekaran et al., 2019; Ding et al., 2019), but their low earth abundance and high cost inhibits their practical applicability. In order to address these issues researchers have proposed alternative approaches including replacing the precious metals with non-noble metals or producing metal free systems with high stability and cost effectiveness.

Literature shows that bismuth (Bi) and Bi_2_O_3_ films, nanoparticles and hybrids show excellent electrocatalytic activity toward 4-NP (Hutton et al., 2004; Lezi et al., 2014; Xia et al., 2014). This is because Bi and Bi_2_O_3_ electrocatalysts shows a high surface area, low band gap, and have good chemical and electrochemical properties. Additionally to this, Bi based systems have been shown to be1 good electrochemical sensors for heavy metals, biomolecules, drugs and for the electrocatalytic reduction of nitrates (Zhang et al., 2009; van der Horst et al., 2016; Sakthivel et al., 2019). These merits of Bi motivate researchers to use it for the electrochemical sensing of 4-NP. Bi proved to be a good alternative for precious metals as it is cost effective, sensitive, environmental friendly, versatile, and has a variety of uses (Hutton et al., 2004; Dortsiou and Kyriacou, 2009; Švancara et al., 2010; Wang W. et al., 2019). A Bi_2_O_3_ multiwalled carbon nanotube (Bi2O_3_@MWCNT) composite might provide synergistic effects for the enhanced electrochemical detection of 4-NP. To date, and to the best of our knowledge, Bi_2_O_3_@MWCNTs has not been reported for 4-NP detection and quantification.

## Experimental

### Chemicals

MWCNTs 99.99%, Ethylene glycol (EG) 99.97%, Sulphuric acid 98%, Nitric acid 78%, Bismuth pentahydrate [Bi(NO_3_)_3_ (H_2_O)_5_], Acetone 99.99% and 4-NP (Para-nitrophenol) were purchased from Alfa-Aesar. All the chemicals were used as received for the synthesis of electrocatalysts and electrochemical studies were carried out in deionized water.

### Characterization

X-ray diffraction (XRD) was carried out using a Rigaku Ultima IV fully automatic high-resolution X-ray diffractometer, with the X-ray generator operating at 40 kV and 40 mA at a step of 0.01(2θ) at room temperature. FTIR spectra were measured in the 4,000–400 cm^−1^ range on a Perkin-Elmer Spectrum-I spectrometer with samples prepared as KBr pellets. Raman spectroscopy was performed using a microscope with Raman optics (Seki Technetronic Corporation, Tokyo) with a 532 nm LASER.

### Electrochemical Measurements

All electrochemical studies were performed on a CHI660C electrochemical works station (CH-Instrument) with a three electrode system. A glassy carbon (GC, 3 mm dia.) electrode was used as the working electrode to support the catalysts. A Pt foil and saturated SCE were used as the counter and reference electrodes, respectively. The GC electrode was polished with three different sizes of Al_2_O_3_ powder (1, 0.3, and 0.05 μm) followed by cleaning in an ultrasonic bath and finally rinsing with deionized water followed by ethanol. To prepare the working electrode, 0.5 mg as-synthesized catalyst was dispersed in a calculated amount (2–6 mL) of isopropyl alcohol under ultrasonic stirring for 40 min. An aliquot of the slurry was dropped onto the pre-polished GC electrode by using a micropipette and dried under an infrared lamp. The loading of catalyst on the electrode was calculated and used for normalization of the current.

### Synthesis of Acid Functionalized Multiwalled Carbon Nanotubes (MWCNTs)

In a typical synthesis, 1 g of MWCNT powder was added to 120 mL nitrating mixture (30 mL HNO_3_ and 90 mL H_2_SO_4_) in a round bottom flask which was placed in an ice bath under stirring for 30 min. Then mixture was then subjected to ultrasonication at room temperature for 2 h. After sonication the dispersion was refluxed for 6 h, and a brown-gray paste was obtained after completion of the reaction (defined as diminished effervesence). After separating it *via* centrifugation, the brown-gray paste was re-dispersed in deionized (DI) water and subjected to ultrasonication for 3 h. After filtration the obtained solid material was washed with 1 M HCl, followed by an adequate amount of DI water until it turned into a black powdery material. These MWCNTs were used for further characterization and doping with bismuth.

### Synthesis of Bi_2_O_3_ Decorated Multiwalled Carbon Nanotubes and Bi_2_O_3_ NPs

**Scheme 1:**
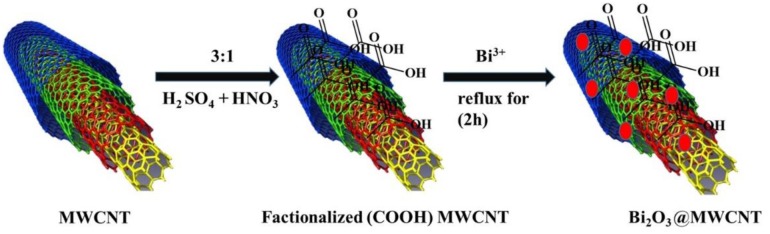
Oxidative functionalization of MWNTs followed by decoration with Bi_2_O_3_ NPs.

In a typical synthesis, 50 mL of ethylene glycol (EG) was heated at 110 °C for 30 min under constant stirring to remove dissolved oxygen and water molecules. This obtained anhydrous EG was used for further synthesis. Following this, 20 mg of acid functionalized MWCNTs were added to 50 mL anhydrous EG and dispersed by sonication for 3 h. On complete dispersion of the MWCNTs, bismuth nitrate (0.02 gm in 50 mL EG) was added dropwise under constant stirring and stirring continued for 3 h. Then this reaction mixture was refluxed for 6 h. The resultant product was cooled to RT then filtered and washed with acetone. The obtained black colored catalyst was dried in the oven at 60°C for 2 h which results in Bi_2_O_3_@MWCNTs nanocomposite formation and is demonstrated schematically in Scheme 1. Similarly, Bi_2_O_3_ NPs were synthesized without the addition of MWCNTs.

## Results and Discussion

**Figure 1:**
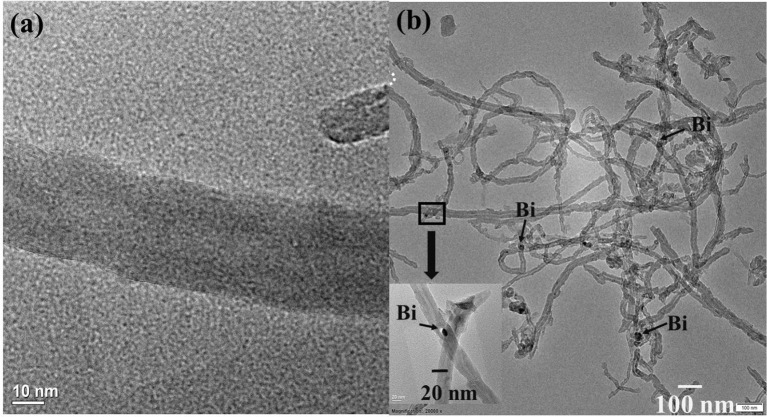
**(a)** TEM image of MWCNTs showing that the average diameter is 10 nm, **(b)** TEM image of Bi_2_O_3_@MWCNTs showing that the average size of Bi_2_O_3_@MWCNTs is ~20 nm.

**Figure 2:**
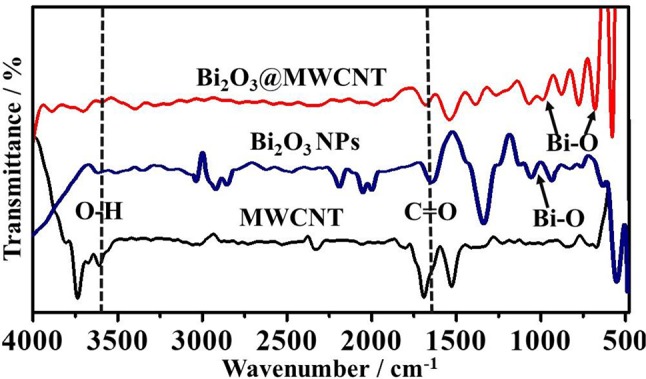
Superimposed FTIR spectra of MWCNTs, Bi_2_O_3_ NPs and Bi_2_O_3_@ MWCNTs. Acid functionalization followed by Bi_2_O_3_ decoration is confirmed by the appearance of additional signals corresponding to Bi-O.

The morphological and structural features of electrocatalysts were identified by TEM and are shown in Figure 1a. The TEM image of the MWCNTs shows the average diameter is ~10 nm. Moreover, Figure 1b shows the TEM for Bi_2_O_3_@MWCNTs. MWCNTs were found to provide a large surface area for crystalline Bi NPs with spherical shape. Further, the superimposed FTIR spectrum of MWCNTs, Bi_2_O_3_ NPs and Bi_2_O_3_@MWCNTs is displayed in Figure 2 and it clearly shows bands appearing at 1,500, 1,700, and 3,600 cm^−1^ which correspond to the stretching frequencies of oxygen in C=O, aromatic C=C and O-H, respectively, all of which are functional groups present on MWCNTs.

**Figure 3:**
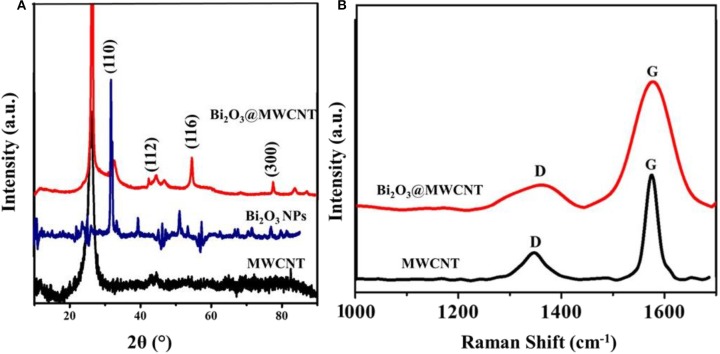
**(A)** X-ray diffraction (XRD) pattern of MWCNTs, Bi_2_O_3_ NPs and the Bi_2_O_3_@MWCNTs nanocomposite and **(B)** Raman spectra of MWCNTs and the Bi_2_O_3_@MWCNTs nanocomposite.

Bi_2_O_3_@MWCNTs clearly indicates that the peak around 600 cm^−1^ and 1,000 cm^−1^ corresponds to Bi-O stretching frequencies (Chipeture et al., 2019). The XRD (crystal structure) images of MWCNTs and Bi_2_O_3_@MWCNTs is displayed in Figure 3A and shows sharp signals for Bi_2_O_3_ NPs and MWCNTs in the 2θ of range 10 to 90°. The characteristic diffraction patterns (002) and (101) correspond to the MWCNTs, which closely agree with reported functionalized MWCNTs systems. Moreover, Bi_2_O_3_ has an α-metastable crystal structure that was confirmed by corresponding peaks observed at the (110), (116), and (300) planes. Raman spectra for MWCNTs and the Bi_2_O_3_@MWCNTs composite are shown in Figure 3B. In case of MWCNTs a signal appeared corresponding to the D band at 1,345 cm^−1^ and the G band at 1,570 cm^−1^ and their associated intensity ratio (i.e., I_D_/I_G_) ratio is 0.32. For Bi_2_O_3_@MWCNTs the calculated intensity ratio, I_D_/I_G_, is 0.88. The increase of I_D_/I_G_ by more than double confirms an increase in the disorder in MWCNTs after decoration with Bi_2_O_3_ NPs. It is in good agreement with previous reports (Thi Mai Hoa, 2018).

**Figure 4:**
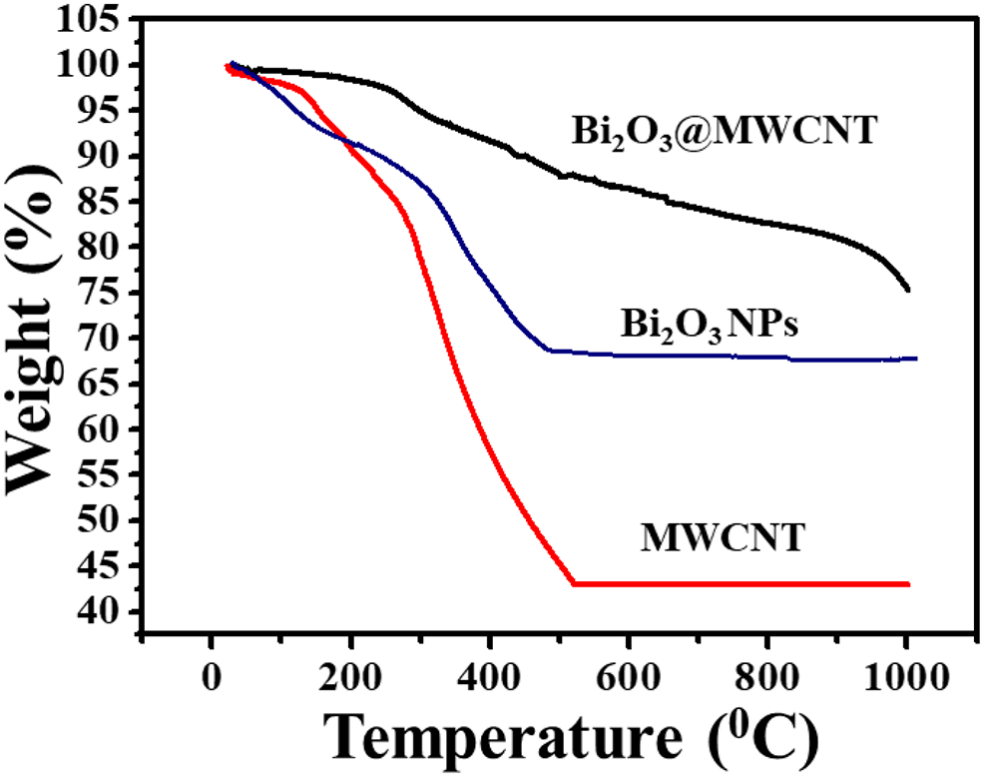
Superimposed TGA profiles for MWCNTs, Bi_2_O_3_ NPs and the Bi_2_O_3_@MWCNTs nanocomposite in an air atmosphere.

Thermogravimetric analysis deals with weight loss during decomposition as a function of temperature, in the range of 0–1,000°C. In case of MWCNTs the degree of functionalization, i.e., the extent to which carboxylic groups were grafted onto MWCNTs, was confirmed by TGA. Figure 4 shows the superimposed TGA curves for MWCNTs, Bi_2_O_3_ NPs and Bi_2_O_3_@MWCNTs. In the case of MWCNTs (red line) a two-step decomposition has been observed, in the first step, which ranges from 100 to 300°C, ~ 23% of weight was lost due to the loss of water molecules and other volatile species. In the second stage, ranging between 300 and 520°C, rapid weight loss was observed to give an overall weight loss of 58%, this could be due to functional groups like –COOH and is in good agreement with the literature (Thi Mai Hoa, 2018). In the case of Bi_2_O_3_ NPs (blue line) a two-step weight loss is observed. The first step between 70 and 270°C corresponds to 13% weight loss due to the elimination of water and other impurities. While in the second step, after 270°C, an overall 33 % weight loss is observed. This is due to the low melting point of Bi (271°C) and the removal of capping molecules. When compared to MWCNTs & Bi_2_O_3_ NPs, the Bi@MWCNTs nanocomposite (black line) shows much more thermal stability. It shows a rapid decomposition after 271°C which is attributed to the low melting point of bismuth.

### Electrochemical Studies Toward 4-Nitrophenol as an Organic Pollutant

**Figure 5:**
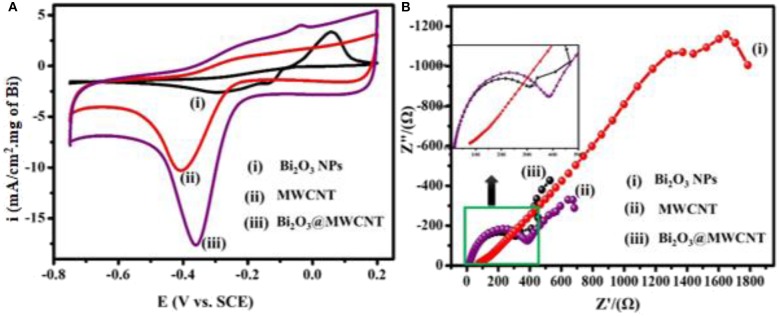
**(A)** CV of (i) Bi_2_O_3_ NPs (ii) MWCNTs (iii) Bi_2_O_3_@MWCNTs in the presence of 4 mM 4-NP in 0.5M H_2_SO_4_ at 50 mV/s, **(B)** Nyquist plots for (i) Bi_2_O_3_ NPs (ii) MWCNTs (iii) Bi_2_O_3_@MWCNTs in the presence of 4 mM 4-NP in 0.5 M H_2_SO_4_ at 50 mV/s (current density is normalized with loading of Bi_2_O_3_ NPs in Bi_2_O_3_@ MWNTs calculated from TG analysis).

The electrochemical behavior of as-synthesized electrocatalytic systems was investigated by using cyclic voltammetry (CV) in 0.5 M H_2_SO_4_ as a supporting electrolyte. Figure 5A shows the CV response of Bi_2_O_3_ NPs, MWCNTs, and Bi_2_O_3_@MWCNTs in the presence of 4-NP (4 mM) in 0.5 M H_2_SO_4_ at a scan rate of 50 mV/s. It has been observed that Bi_2_O_3_ NPs show the smallest reduction peak but MWCNTs shows a slightly higher reduction peak. In the case of Bi_2_O_3_@MWCNTs a significant increase in reduction peak was observed which confirms the synergistic effect of Bi_2_O_3_ and MWCNTs, i.e., due to increases in the electron density of Bi and the higher accessible surface of MWCNTs toward 4-NP sensing.

Electrochemical Impedance Spectroscopy (EIS) is an efficient tool which provides insights about the interfacial picture during electron transfer at interfaces (Wu et al., 2018) during the reductive sensing of 4-NP. In EIS studies the diameter of a semicircle is directly proportional to electron transfer resistance which governs the electron transfer kinetics (He et al., 2019). Figure 5B shows Nyquist plots for Bi_2_O_3_ NPs, MWCNTs and Bi_2_O_3_@MWCNTs in 0.5 M H_2_SO_4_. The larger semi-circle observed for Bi_2_O_3_ NPs and MWCNTs reflects a higher electron transfer resistance. On the contrary Bi_2_O_3_@MWCNTs (309 Ω) shows a small semi-circle which reflects a lower electron transfer resistance which results in higher electrocatalytic activity toward 4-NP reduction compared to Bi_2_O_3_ NPs (1,000 Ω) and MWCNTs (400 Ω). These result are in good agreement with cyclic voltammetry results. Table 1 shows comparative data of the onset potential and peak current density of electrocatalysts, it can be seen that the Bi_2_O_3_@MWCNTs nanocomposite has a lower negative onset potential and higher peak current density as compared to Bi_2_O_3_ NPs and MWCNTs, respectively.

**Table 1:** Comparative data of onset potential and peak current density of electrocatalysts.

**Sr.No**.	**Electrocatalyst**	**Onset Potential (V vs. SCE)**	**Current density i (mA/cm^**2**^)**
1	Bi_2_O_3_ NPs	−0.19	2.6
2	MWCNTs	−0.24	10.0
3	Bi_2_O_3_@MWCNTs	−0.17	17.62

**Figure 6:**
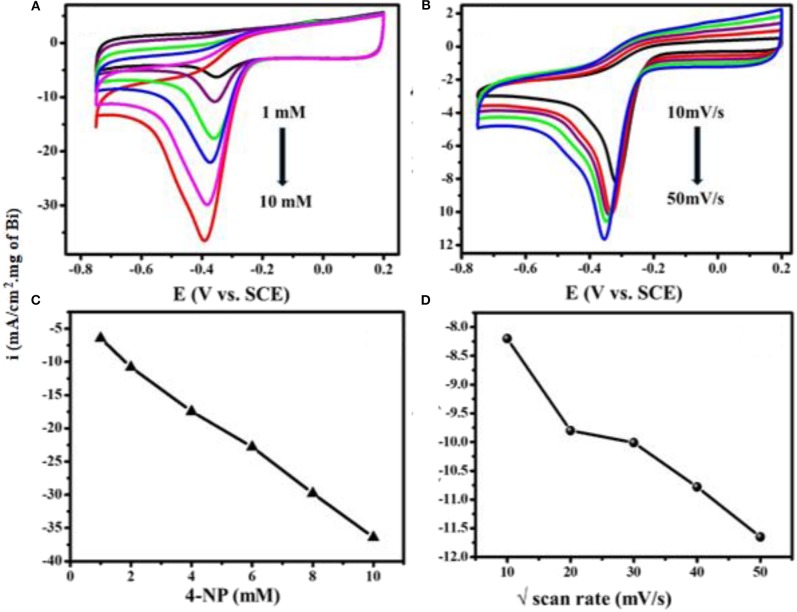
Superimposed CV curves of Bi_2_O_3_@MWCNTs in 0.5 M H_2_SO_4_ **(A)** at different concentrations from 1 mM to 10 mM of 4-NP at a scan rate of 50 mV/s **(B)** at different scan rates (10–50 mV/s) for 4 mM of 4-NP. **(C)** shows the linear fit for peak current density vs. concentration and **(D)** linear fit peak for the scan rate of 4-NP on Bi_2_O_3_@MWCNTs (current density is normalized with loading of Bi_2_O_3_ NPs in Bi_2_O_3_@ MWNTs calculated from TG analysis).

Figure 6A shows superimposed CV curves for different concentrations of 4-NP on Bi_2_O_3_@MWCNTs in 0.5 M H_2_SO_4_ at a scan rate of 50 mV/s. The reduction peak current increases linearly with concentration (1, 2, 4, 6, 8, 10 mM) which means Bi_2_O_3_@MWCNTs shows efficient electrochemical sensing toward 4-NP. Figure 6B shows scan rate dependent studies on the reduction peak current of Bi_2_O_3_@MWCNTs, in 0.5 M H_2_SO_4_ at a concentration of 4 mM. The variations in their linearity with current density and positive shift in potential could be due to diffusion controlled 4-NP reduction processes as shown in Figures 6C,D.

**Figure 7:**
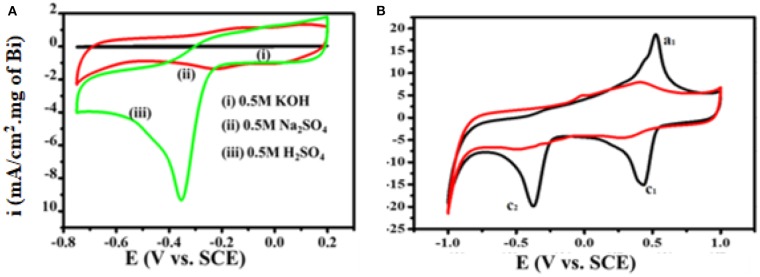
**(A)** Superimposed CV curves for 4-NP sensing in different media **(B)** CV obtained at Bi_2_O_3_@MWCNTs without 4-NP (red line) and Bi_2_O_3_@MWCNTs with 4-NP (black line) in 0.5 M H_2_SO_4_ with a potential window of −1.0 to 1.0, at scan rate 50 mV/s.

Figure 7A shows 4-NP electrochemical sensing by the Bi_2_O_3_@MWCNTs nanocomposite in acidic, basic and neutral media. As electroreduction of 4-NP is a proton involving step, the pH of the supporting electrolyte affects the electrochemical processes. From Figure 7A it is evident that a distinct reduction peak is observed in the case of an acidic medium as compared to neutral and basic media. This is due to the fact that protons replaced the 4-NP present on electrode surface, as the pH of the medium is increased nitrogen anions prevent the approach of 4-NP to the electrode surface (Wu et al., 2018). The mechanism has been proposed for the electrochemical reduction of 4-NP on Bi_2_O_3_@MWCNTs on the basis of CV is shown in Figure 7B. There is not an appreciable peak observed in the absence of 4-NP (red line) while in the presence (black line) of 4-NP the redox couple is observed at Ea_1_= 0.52 and Ec_1_= 0.43 and the reduction peak is at Ec_2_= −0.37 which confirms the reduction of 4-NPs.

The C_2_ peak symbolizes the formation of 4-hydroxylaminophenol which is an irreversible reaction while the redox couple is due to the interconversion of 4-hydroxylnitrophenol and 4-nitrosophenol (Rajkumar et al., 2018; He et al., 2019).

**Figure 8:**
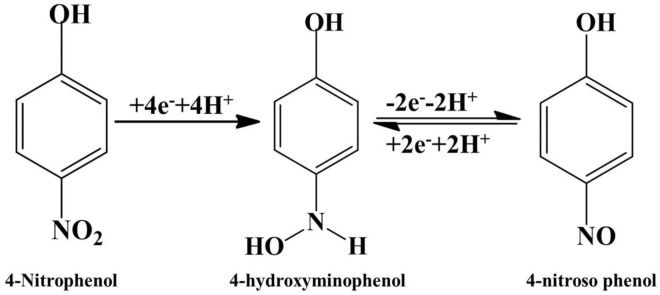
Mechanism of 4-Nitrophenol reduction on Bi_2_O_3_@MWCNTs.

The electrochemical mechanism is given in Figure 8.

### Limit of Detection (4-NP)

The limit of detection is an important parameter for analytical method validation. Figure 6A depicts cyclic voltammetric response for different concentrations of 4-NP vs. peak current densities. Accordingly,

(1) $$\begin{array}{l} LOD=3\left(S/M\right) \end{array}$$

Where, S, Standard deviation; M, Slope point.

The LOD is calculated by using equation 1 and the LOD was calculated to be 0.1 μM (Mulik et al., 2018). Finally it is found that a smaller LOD confirms the actual apparent concentration of the 4-NP reduction reaction. In addition to this, our work has been compared with the reported literature. Table 2 summarizes reported electrocatalytic systems with their linearity range and LOD upon comparison proposed Bi_2_O_3_@MWCNTs system for 4-NP reduction (Mulik et al., 2018).

**Table 2:** Comparison of 4-nitrophenol determination with Bi_2_O_3_@MWCNTs as with other electrocatalysts reported in literature.

**Sr. No**.	**Electrocatalyst**	**Technique**	**Linear range**	**LOD (μM)**	**References**
1	S-GCN/SPCE	i-t	0.05–90 μm	**-**	Rajkumar et al., 2018
2	β -CD/SiC/GCE	i-t	5–150 μM	**-**	Wu et al., 2018
3	Cu-Curcumin/GCE	DPV	0.1–1,030 μM	**-**	Bose and Ramiah, 2017
4	Ag NPs –decorated TA@Fe_3_O_4_/GCE	DPV	0.1–680 μM	**-**	Sangili et al., 2018
5	Au@MWCNTs/GCE	i-t	1 × 10^−8^ to 5 × 10^−4^ M	**-**	Al-Kahtani et al., 2018
6	SWCNT/GCE	i-t	1 × 10^−8^ to 5 × 10^−6^ M	-	Yang, 2004
7	FeOx/TiO_2_@mC/GCE	CV	5–310μM	0.183	Wang M. et al., 2019
8	α- MnO_2_/MWCNTs	CV	30–475 μM	0.186	Anbumannan et al., 2019
9	2D ZnCo_2_O_4_ Nanosheets	DPV	1–4,000 μM	0.3	Zhang et al., 2018
10	DTD/AgNPs/CPE	CV	1–100	0.25	Rounaghi et al., 2011
11	RGO/Fe_3_O_4_NPs/GCE	DPV	0.2–10 μM	0.26	Cheng et al., 2017
		SWV	20–100 μM	0.86	
12	Cu_2_O Sheets	CV	0.006-2.72 μM	0.5	Veeramani et al., 2016
13	GNFs/GCE	CV	1–6000	0.7	Wang et al., 2014
14	Bi_2_O_3_@MWCNTs	CV	1-10 mM	0.10	This work

## Conclusion

The Bi_2_O_3_@MWCNTs electrocatalyst was synthesized by using a simple chemical reduction method. As-synthesized nanomaterials MWCNTs, Bi_2_O_3_ and Bi_2_O_3_@MWCNTs NPs have been well-characterized by FTIR which confirms the Bi-O bonding in Bi_2_O_3_@MWCNT. XRD shows the Bi_2_O_3_@MWCNTs was in an α-metastable crystal structure. Raman spectra show the I_D_/I_G_ ratio increases in Bi_2_O_3_@MWCNTs as compared with MWCNTs, and it is confirmed that there are more sp^2^ C, TEM analysis confirms the average size is ~10 nm. The improved monometallic Bi supporting MWCNTs provides a higher surface area which results in a significant increase in the electrocatalytic activity with an onset potential of −0.17 V toward electrochemical 4-NP reduction. As compared to reported systems, this is a cost effective and highly efficient system for the determination of 4-NP.

## Data Availability Statement

The datasets generated for this study are available on request to the corresponding author.

## Author Contributions

RD has conducted all experiments and written the manuscript. AM and BM have helped to interpret the characterization data, whereas BS has invigilated the whole project with his expert advice and fruitful suggestions.

## Conflict of Interest

The authors declare that the research was conducted in the absence of any commercial or financial relationships that could be construed as a potential conflict of interest.
